# Association of *MBL2* Exon 1 Polymorphisms With Multibacillary Leprosy

**DOI:** 10.3389/fimmu.2020.01927

**Published:** 2020-09-03

**Authors:** Bruna Tiaki Tiyo, Evelyn Castillo Lima Vendramini, Victor Hugo de Souza, Cristiane Maria Colli, Hugo Vicentin Alves, Ana Maria Sell, Sylmara Bessani Paixão Zucoloto, Jeane Eliete Laguila Visentainer

**Affiliations:** ^1^Laboratory of Immunogenetics, Department of Basic Health Sciences, Maringá State University (UEM), Maringá, Brazil; ^2^Inter-municipal Public Health Consortium (CISAMUSEP), Maringá, Brazil

**Keywords:** genetic polymorphism, mannose-binding lectin, multibacillary, genetic predisposition to disease, case–control study, gene frequencies

## Abstract

Mannose-binding lectin (MBL) is a serum protein of innate immunity, with a central role in the activation of the complement system through the lectin pathway. This protein is encoded by *MBL2* gene, and single-nucleotide polymorphisms located at exon 1, such as rs5030737 C>T (*D* variant), rs1800450 G>A (*B* variant), and rs1800451 G>A (*C* variant), may change the MBL structure and the serum concentration. *MBL2* polymorphisms have been associated with several infectious diseases, including leprosy. Host immune response has a major impact on the clinical manifestation of leprosy since only a few individuals infected with *Mycobacterium leprae* will develop the disease. Therefore, the aim of this study was to evaluate the influence of *MBL2* exon 1 polymorphisms (rs5030737, rs1800450, and rs1800451) on the MBL levels and leprosy immunopathogenesis. This case–control study included 350 leprosy patients from Southern Brazil, with 279 classified as multibacillary (MB) and 71 as paucibacillary (PB). The control group consisted of 350 non-consanguineous individuals, who were not diagnosed with leprosy or other infectious and autoimmune diseases. Genotyping was performed by PCR–sequence specific primers, and the MBL serum concentrations were evaluated by ELISA. *MBL2* exon 1 polymorphisms were analyzed individually and grouped as genotypes, considering “A” as the wild allele and “O” as the presence of at least one polymorphism (*D, B*, or *C* variants). Differences were not observed in the distribution of genotypic and allelic frequencies between leprosy *per se* patients and controls. However, in a haplotypic analysis, the TGG haplotype presented a risk for development of leprosy *per se* in women when compared to the wild haplotype (CGG) (OR = 2.69). Comparing patients with MB and PB, in a multivariate analysis, the *B* variant was associated with the susceptibility of developing the MB form of leprosy (OR = 2.55). Besides that, the CAG haplotype showed an increased susceptibility to develop MB leprosy in women compared to men. It was observed that the A/O genotype in women was associated with a susceptibility to leprosy development *per se* (OR = 1.66) and progression to MB leprosy (OR = 3.13). In addition, the MBL serum concentrations were in accordance with the genotyping analysis. In summary, our data suggest that *MBL2* exon 1 polymorphisms are associated with an increased risk to leprosy development and progression.

## Introduction

Mannose-binding lectin (MBL) is a soluble protein responsible for activating the complement system *via* the lectin pathway. In addition, MBL is involved in microorganism opsonization for phagocytosis and macrophage activation (1, 2).

*MBL2* gene, which encodes MBL, is located on chromosome 10 (q11.2-q21) (1). Three variants are commonly studied on *MBL2* exon 1: rs5030737 (g.52771482G>A, p.Arg52Cys), rs1800450 (g.52771475C>T, p.Gly54Asp), and rs1800451 (g.52771466C>T, p.Gly57Glu), also described as *D, B*, and *C* variants, respectively, in contrast to the wild type, which is termed as *A* allele (3, 4). These single-nucleotide polymorphism (SNPs) are known as structural variants since they modify the structure of the protein and the assembly of MBL oligomers, leading to the formation of smaller non-functional oligomers (5). This affects binding avidity, with a possible functional implication, since the prolonged interaction can facilitate self-activation of MASP1 to activate the complement cascade more efficiently (6). Another consequence of MBL variants is increased susceptibility to degradation by metalloproteases (7).

High MBL levels may facilitate the infection of intracellular pathogens into host cells through C3b receptors. Some studies have shown that MBL deficiency indicated protection in diseases such as leishmaniasis (8, 9), tuberculosis, and leprosy (10–12).

Leprosy is a chronic infectious disease caused mainly by *Mycobacterium leprae* and *Mycobacterium lepromatosis* (13, 14), which mainly affect the skin, peripheral nerves, upper respiratory tract mucosa, and eyes (13, 15). According to the World Health Organization (WHO) classification (1982), leprosy patients are considered paucibacillary (PB), when they present up to five cutaneous lesions, or multibacillary (MB), which includes patients with more than five lesions (13).

Regarding the epidemiology of leprosy, at the end of 2018, WHO reported an incidence of 2.74 new cases per 100,000 (208,619 cases). More than 79% of new cases refer only to three countries: India, Brazil, and Indonesia. In this case, Brazil was the second country with the largest number of new leprosy cases in the world, totaling 28,660 (16, 17).

Host immune response has a major impact on the clinical manifestation of leprosy, and according to some studies, there is an association between MBL protein and the development and the progression of this disease (4, 18–20). Thus, the objective of this work was to evaluate the influence of *MBL2* gene exon 1 polymorphisms (*D, B*, and *C* variants), which encodes the MBL protein, on leprosy immunopathogenesis in residents of the north/northwest regions of Paraná, Brazil.

## Materials and Methods

### Study Population

This case–control study consisted of leprosy patients and controls. The leprosy patients were classified following the WHO classification, multibacillary and paucibacillary. All the participants were residents of the northern and the northwestern regions of Paraná, southern Brazil (22°29′30″-26°42′59″ S and 48°02′24″-54°37′38″W). The population was considered as mixed according to Probst et al. (21) who described the Paraná ethnic constitution as predominantly of European origin (80.6%), with a small contribution from African (12.5%) and Amerindian (7.0%) ancestry (21).

The control group in this study was composed of both non-consanguineous individuals who were in household contact with leprosy patients and individuals without previous contact with these patients. All the control subjects declared that they did not present leprosy or other infectious and autoimmune diseases. The leprosy patients were diagnosed by clinical examination, bacilloscopy, and biopsy and classified by experienced dermatologists at the CISAMUSEP (Inter-municipal Public Health Consortium).

All the individuals who accepted to participate in the research signed the informed consent form, previously approved by the State University of Maringá (UEM) Human Research Ethics Committee (2.424.046/2017).

### Genotyping and Quantification of Serum MBL

Genomic DNA was extracted from peripheral blood using BIOPUR® commercial kit (Mobius Life Science, Curitiba, Paraná, Brazil) according to the manufacturer's recommendations. Subsequently, three *MBL2* exon 1 SNPs were genotyped as *D* (rs5030737), *B* (rs1800450), and *C* (rs1800451) variants by polymerase chain reaction–sequence-specific primers (PCR–SSP) according to Steffensen et al., with modifications (22). The reactions were performed in a volume of 10 μl, containing 2 ng/μl of each primer, 1X buffer, 1.0 mM MgCl_2_ for mix D, ABC, and ACD, and 1.5 mM MgCl_2_ for mix B, C, and ABD, 0.2 mM dNTP, GoTaq® Flexi DNA Polymerase 1U (Promega Corporation, Madison, WI, USA), and 100 ng DNA. The positive controls used in the PCR–SSP reactions were previously characterized by genomic sequencing.

DNA amplification was performed in an Applied Biosystems® Veriti Thermal Cycler (Thermo Fisher Scientific, Foster City, CA, USA). The cycling conditions used were 95°C for 10 min, followed by 30 cycles of 94°C for 20 s, 65°C for 20 s, 72°C for 30 s, and a final extension of 72°C for 5 min. For mix C, the annealing temperature was 68°C. Detection of amplified fragments was performed by DNA electrophoresis on 2% agarose gel with SYBR® Safe® (Invitrogen Life Technologies, Grand Island, NY, USA) and visualized in ultraviolet light. The primers that amplify the growth gene region (HGH) were used as internal reaction controls (22).

Quantification of serum MBL was performed using a commercial capture enzyme-linked immunosorbent assay (MBL Oligomer ELISA kit; BIOPORTO® Diagnostics, Hellerup, Denmark), according to the manufacturer's instructions. The serum or plasma of patients was diluted 1:100. Absorbances were read at 450 nm in a FlexStation® 3 Multi-Mode microplate reader (Molecular Devices, San Jose, CA, USA).

### Statistical Analysis

Allelic, genotypic, and haplotypic frequencies were calculated for patients and controls, and Hardy–Weinberg equilibrium (HWE) was evaluated from a genotype distribution analysis. Multiple SNP comparisons were performed for *MBL2* exon 1 SNPs. Haplotypic analysis was performed for three *MBL2* exon 1 SNPs as well as the linkage disequilibrium. For the analysis of multiple SNPs, the software Haploview (23) was used. Through multiple SNP analyses, we estimated the frequency of possible haplotypes using the implementation of expectation–maximization algorithm. This analysis allows one to check if these haplotypes demonstrated an association with leprosy, including linkage disequilibrium (LD).

The allelic, genotypic, and haplotypic frequencies of the studied polymorphisms, as well as the association analyses between genetic polymorphisms and leprosy, were obtained by the SNPStats program (available at: https://www.snpstats.net/) (24). The genetic inheritance models considered were codominant, dominant, recessive, overdominant, and log additive. The best inheritance model was chosen based on the lowest value for the Akaike information criterion (AIC). Odds ratio (OR) and 95% confidence interval (CI) were calculated by logistic regression tests, after including variables such as gender and age. Logistic regression test was used to verify the association of SNPs in leprosy progression. *P* < 0.05 was considered as statistically significant. Bonferroni correction for the total number of SNPs was not required by the reason that exon 1 had a high LD.

The patient and the control groups were matched for gender and age variables. In order to obtain the minimum number of samples adequate to carry out this study with adequate statistical power (≥ 80%), the quantitative calculation software QUANTO (25) was used, which takes into account the frequencies of SNPs in the population and the prevalence of the disease (leprosy *per se*). Besides that, the sample size was calculated after consideration of the minor allele frequency.

Another analysis performed in this work was grouping the three SNPs (*D, B*, and *C* variants) into a single allele, represented by the letter “O,” with the wild allele as “A” (Table 1).

**Table 1:** *MBL2* exon 1 grouped genotypes.

**Grouped genotypes**	**Genotypes for each grouped genotype**
*A/A*	C/C (*D* variant), G/G (*B* variant), and G/G (*C* variant)
*A/O*	C/T (*D* variant), G/A (*B* variant), and G/A (*C* variant)
*O/O*	T/T (*D* variant), A/A (*B* variant), A/A (*C* variant), C/T+G/A (*D* and *B* variants), C/T+G/A (*D* and *C* variants), and G/A+G/A (*B* and *C* variants)

The serum levels of MBL were calculated with a four-parameter logistic curve using My Curve Fit (https://mycurvefit.com/), with a dilution factor of 1:100. The MBL levels were compared within gender and age of patients with Pearson correlation coefficient and also between PB and MB patients with Student's *t*-test. The concentrations correlated to polymorphisms were evaluated separately (*D, B*, and *C*) or grouped (*A* and *O*) using Mann–Whitney *U* test in R software, version 3.5.2., by the reason that we had evaluated the normality by Shapiro–Wilk test, and this test indicated the need of a non-parametric test. For all analyses, *P* < 0.05 was considered as statistically significant.

## Results

We included 350 leprosy patients (193 males and 157 females, mean age 54 ± 13 years) and 350 controls (180 males and 170 females, mean age 56 ± 13 years) in the study. Of the leprosy patients, 279 (79.7%) were MB and 71 (20.3%) were PB (Supplementary Table 1).

For all genotypic analyses, the codominant association model was considered, which allows each genotype to present a different and non-additive risk. However, for the *B* variant, the log-additive (or multiplicative) model was used, which considers that each mutated allele modifies the risk in an additive form, that is, a homozygous individual for a mutated allele has a double risk compared to a heterozygous one to the same allele. The best inheritance model was chosen based on the lowest value for the AIC (https://www.snpstats.net/tutorial.htm).

Differences were not observed in the distribution of genotypic and allelic frequencies between leprosy *per se* patients and controls (Table 2). However, in haplotypic analysis, TGG haplotype was associated with a susceptibility to the development of leprosy *per se* in women (OR = 2.69, 95% CI = 1.04–6.97) when compared to the wild haplotype (CGG) (Table 3). Among the *MBL2* gene SNPs analyzed in this study, the most frequent mutated allele was codon 54 (*B* variant) in both patients (total and their classifications) and controls. The distribution of genotypes for all SNPs was in HWE. The statistical power obtained at 0.05 level of significance, two-tailed test, *K*_P_ = 0.0025, for the *D, B*, and *C* variants were power values of 93.7, 99.9, and 98.8%, respectively, to detect a genetic effect of 2.5 under a dominant model.

**Table 2:** Genotype and allele frequency distributions for *MBL2* exon 1 polymorphisms in leprosy *per se*, paucibacillary (PB) and multibacillary (MB) patients and controls.

**SNP**	**Allele/** **genotype**	**Leprosy patients** **(*****N*** **=** **350)**			**Controls** **(*N* = 350)**
		***Per se*** **(*N* = 350)**	**MB** **(*N* = 279)**	**PB** **(*N* = 71)**	
		***n* (%)**	***n* (%)**	***n* (%)**	***n* (%)**
Codon 52	C	661 (94%)	528 (95%)	133 (94%)	669 (96%)
(rs5030737)	T	39 (6%)	30 (5%)	9 (6%)	31 (4%)
*D* variant	C/C	312 (89.1%)	249 (89%)	63 (89%)	319 (91%)
	C/T	37 (10.6%)	30 (11%)	7 (10%)	31 (9%)
	T/T	1 (0.3%)	0	1 (1%)	0
Codon 54	G	614 (88%)	482 (86%)	132 (93%)	617 (88%)
(rs1800450)	A	86 (12%)	76 (14%)	10 (7%)	83 (12%)
*B* variant	G/G	269 (77%)	208 (74.5%)	61 (86%)	270 (77%)
	G/A	76 (22%)	66 (23.5%)	10 (14%)	77 (22%)
	A/A	5 (1%)	5 (2%)	0	3 (1%)
Codon 57	G	655 (94%)	522 (94%)	133 (94%)	658 (94%)
(rs1800451)	A	45 (6%)	36 (6%)	9 (6%)	42 (6%)
*C* variant	G/G	308 (88%)	245 (88%)	63 (89%)	310 (88.5%)
	G/A	39 (11%)	32 (11%)	7 (10%)	38 (11%)
	A/A	3 (1%)	2 (1%)	1 (1%)	2 (0.5%)

*SNP, single-nucleotide polymorphism; N, number of subjects; n, number of alleles or genotypes (frequency)*.

**Table 3:** Haplotypic frequencies of *MBL2* exon 1 polymorphisms in leprosy patients and controls within gender.

**Haplotype**	**Frequency^**a**^**	**Female** **OR (95% IC)**	**Male** **OR (95% IC)**
CGG	0.77	Ref.	Ref.
CAG	0.12	1.52 (0.91–2.52)	0.84 (0.53–1.35)
CGA	0.06	1.27 (0.68–2.34)	1.01 (0.56–1.83)
TGG	0.05	2.69 (1.04–6.97)	0.96 (0.49–1.88)

*OR, odds ratio (95% IC); Ref., reference*.

a *Estimated relative frequency for each haplotype. Haplotype association is evaluated by logistic regression, and the most frequent haplotype is chosen automatically. The risk for each haplotype is compared with the reference, which is the most frequent haplotype (24)*.

Although not associated with the disease, the allelic variants were in linkage disequilibrium. In the multivariate analysis, when comparing MB and PB patients, it was observed that patients with *B* variant were more susceptible to the development of MB leprosy (OR = 2.55, 95% CI = 1.24–5.24) (Table 4). For these analyses, the statistical power for the less frequent allele was above 80% for a risk effect of 4.5, while for the most frequent allele (*B* variant) the risk effect was 2.5 in the log-additive model. In a haplotypic analysis, the CAG haplotype was associated with a susceptibility to the development of MB leprosy in women (OR = 2.69, 95% CI = 1.04–6.97) when compared to the wild haplotype CGG (Table 5).

**Table 4:** Genotypic frequency for *B* variant (rs1800450), located at codon 54 of exon 1 of *MBL2* gene, among multibacillary (MB) and paucibacillary (PB) leprosy patients.

**Association model**	**Genotype**	**PB patients**	**MB** **patients**	**OR** **(95% IC)**	***P*-value**	**AIC**
Codominant	G/G	61 (85.9%)	208 (74.5%)	Ref.	0.017	335.1
	G/A	10 (14.1%)	66 (23.7%)	2.40 (1.14–5.06)		
	A/A	0 (0%)	5 (1.8%)	NP		
Dominant	G/G	61 (85.9%)	208 (74.5%)	Ref.	0.008	334.2
	G/A–A/A	10 (14.1%)	71 (25.4%)	2.56 (1.22–5.37)		
Recessive	G/G–G/A	71 (100%)	274 (98.2%)	Ref.	0.14	339.1
	A/A	0 (0%)	5 (1.8%)	NP		
Overdominant	G/G–A/A	61 (85.9%)	213 (76.3%)	Ref.	0.018	335.6
	G/A	10 (14.1%)	66 (23.7%)	2.34 (1.11–4.93)		
Log additive	–	–	–	2.55 (1.24–5.24)	0.0056	333.6

*SNP, single-nucleotide polymorphism; OR, odds ratio (95% IC); ref., reference; NP, not performed; AIC, Akaike information criterion*.

**Table 5:** Haplotypic frequencies of *MBL2* exon 1 polymorphisms in multibacillary (MB) and paucibacillary (PB) leprosy patients within gender.

**Haplotype**	**Frequency^**a**^**	**Female** **OR (95% IC)**	**Male** **OR (95% IC)**
CGG	0.77	Ref.	Ref.
CAG	0.12	2.69 (1.04–6.97)	2.62 (0.61–11.28)
CGA	0.06	1.55 (0.57–4.23)	0.74 (0.24–2.29)
TGG	0.05	1.47 (0.50–4.29)	0.52 (0.16–1.76)

*OR, odds ratio (95% IC); ref., reference*.

a *Estimated relative frequency for each haplotype. Haplotype association is evaluated by logistic regression, and the most frequent haplotype is chosen automatically. The risk for each haplotype is compared with the reference, which is the most frequent haplotype (24)*.

Another analysis was performed by grouping the three SNPs (*D, B*, and *C* variants) into a single allele. It was observed that the *A/O* genotype in women was associated with a susceptibility to leprosy development *per se* (OR = 1.66, 95% CI = 1.04–2.63) (Table 6) and progression to MB leprosy (OR = 3.13, 95% CI = 1.45–6.75) (Table 7).

**Table 6:** Genotypic frequency of *MBL2* exon 1 polymorphisms, analyzed by grouped genotypes, between female leprosy patients and female controls.

**Genotype**	**Controls**	**Leprosy *per se***	**OR (95% IC)**
*A/A*	109	82	Ref.
*A/O*	53	66	1.66 (1.04–2.63)
*O/O*	8	9	1.58 (0.58–4.30)

*OR, odds ratio (95% IC); ref., reference*.

**Table 7:** Genotypic frequency of *MBL2* exon 1 polymorphisms, analyzed by grouped genotypes, between female MB and PB leprosy patients.

**Genotype**	**PB patients**	**MB patients**	**OR (95% IC)**
*A/A*	33	49	Ref.
*A/O*	12	54	3.13 (1.45–6.75)
*O/O*	3	6	1.24 (0.28–5.37)

*OR, odds ratio (95% IC); ref., reference*.

**Figure 1:**
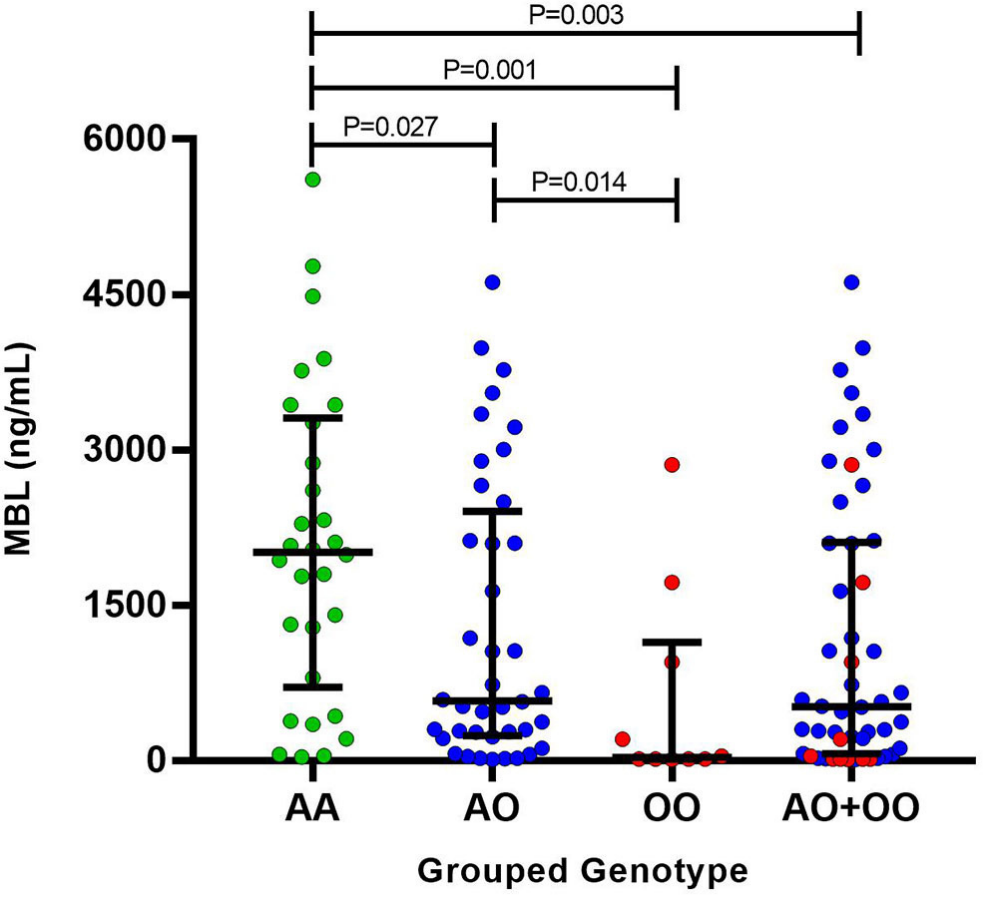
Mannose-binding lectin serum levels between the grouped genotypes.

The serum levels of MBL were evaluated in 80 leprosy patients (Supplementary Table 2). There was no difference when comparing the serum levels of MBL with age and gender. No difference was observed between the serum levels of MBL from MB and PB patients. Comparing the MBL levels with genotypes, statistically significant differences were observed between the grouped genotypes (Figure 1), but not when those were evaluated separately.

## Discussion

The *MBL2* variants have been associated with various diseases, including leprosy (8–12). We evaluated exon 1 polymorphisms (*D, B*, and *C* variants) in leprosy patients and controls from Southern Brazil. In this population, the *B* variant of *MBL2* exon 1 polymorphism was associated with susceptibility in leprosy patients for progression to MB form. Moreover, the grouped genotypes were also related to different MBL levels. Patients with *A/O* and *O/O* compound genotypes presented a decrease in MBL levels compared to the wild genotype (*A/A*). Other significant associations occurred only when we evaluated the female population separately. In a haplotypic analysis, in women, the TGG haplotype was associated with a susceptibility to the development of leprosy *per se*; CAG haplotype was associated with susceptibility for progression to MB as well. In the grouped genotypes, a susceptibility association in female patients was also observed for both the development of leprosy *per se* and progression to MB form.

Even if it is known that men have an increased susceptibility for leprosy (26), we could not find this association. However, when we evaluated the female population separately, we found that the TGG and the CAG haplotypes increased the risk for development of leprosy and progression to MB form, respectively, when compared to women who have wild haplotype. Besides that, the grouped genotype *A/O* was also associated with a risk for development of leprosy and progression to MB form in women. On the other hand, when we evaluated only the male population, we did not find haplotype or genotype as a risk or protection factor. More studies with a greater sample size are necessary to confirm these findings.

In a northwest Brazilian population, Vasconcelos et al. (27) did not observe associations between *MBL2* exon 1 variants and leprosy (27); in contrast, Sapkota et al. (20) observed that the *MBL2 B* variant was associated with protection to the lepromatous form compared to the tuberculoid form of leprosy in a Nepalese population (20). These differences can be explained by population genetics background.

Previous studies have shown that low levels of MBL protein may influence the pathogenesis of diseases that have intracellular microorganisms as an agent (8, 9), such as leprosy (19). The deficiency of this protein has already been associated with protection against the lepromatous form of the disease, also classified as MB (18–20). Despite that we did not point an association between MBL serum levels and the development of the disease, it was observed that the presence of at least one polymorphism (*D, B*, or *C* variants) may decrease the MBL levels. Moreover, the *B* variant of *MBL2* exon 1 polymorphism was associated with susceptibility in leprosy patients for progression to MB. It is well known that there are other polymorphisms in the *MBL2* gene, such as in the promoter and the untranslated regions, and they also can alter the MBL serum concentration and the activity of this protein (1, 5). Although we have not studied these other polymorphisms, this limitation was not crucial in our findings, and we may confirm these results with other polymorphisms in a future study. We did not perform MBL concentration in all samples; however, we equally selected MB and PB patients, with heterogeneous genotyping in both groups. We considered a representative sample of each variant, taking into account variables such as age and gender.

Studies suggest that the binding between MBL and lipoarabinomannan, present on the cell surface of mycobacteria, promotes an increase in phagocyte ingestion of the pathogen (28). Thus, instead of this interaction between MBL and mycobacterium assisting in pathogen elimination, this may increase the uptake and the spread of the pathogen, leading to the establishment of leprosy in its most widespread form, lepromatous (18, 19). In this way, MBL deficiency would have a protective effect against pathogens that use complement-mediated opsonization to enter phagocytes, such as *M. leprae* (18), which also explains the findings of Dornelles et al. (19). Our findings indicate *MBL2* exon 1 polymorphisms to be associated to a reduction in serum protein concentration as a risk for the development of leprosy, especially in the MB form. It is noteworthy that the lectin pathway is not the only complement activation pathway and there are other evasion mechanisms (29).

Moreover, we realized that some patients showed unexpected findings, as we can see in Supplementary Table 2. Some patients had *O/O* genotype, but with high levels of MBL, which may be growing in response to an infection. However, for a complete characterization in high, intermediate, and low concentrations, it is necessary to evaluate other polymorphisms of the *MBL2* gene to correlate each haplotype with its respective classification. Besides that, other patients present *A/A* grouped genotype with a supposedly low concentration of MBL, even though they do not carry an exon 1 *MBL2* polymorphism. Although these findings are surprising, further studies are needed to evaluate other polymorphisms in the *MBL2* gene to better understand the role of MBL in leprosy, and we intend to continue studying these variables. We emphasize that, for all samples which had any confusing factor or bias, we certainly repeated the genotyping.

Finally, the purpose of including a select group of controls, consisting of healthy and non-consanguineous contacts of patients, was to analyze the possible genetic influence on susceptibility or resistance to leprosy (26), considering that this disease develops only in a small group of individuals infected with the bacillus (30). Thus, although these contacts were with the increased possibility of infection, they did not have the disease, suggesting that the absence of infection is due to the immunogenetic factors of each individual.

## Conclusion

We verify the influence of *MBL2* exon 1 polymorphisms on leprosy immunopathogenesis. The presence of the *B* variant was associated with an increased risk of developing multibacillary leprosy, as well as CAG haplotype in women. In addition, the MBL serum concentrations were in accordance with the genotyping analysis, and it was observed that the presence of at least one polymorphism (*D, B*, or *C* variants) may decrease the MBL levels. In summary, our data suggest that *MBL2* exon 1 polymorphisms are associated with an increased risk to leprosy development and progression. For a better understanding of the role of MBL on leprosy, more studies are necessary to evaluate other polymorphisms into the *MBL2* gene, as well as a greater sample size to confirm the findings.

## Data Availability Statement

All datasets presented in this study are included in the article/Supplementary Material.

## Ethics Statement

The studies involving human participants were reviewed and approved by Human Research Ethics Committee of State University of Maringá (UEM). The patients/participants provided their written informed consent to participate in this study.

## Author Contributions

BT and EV conceived the study. BT, CC, EV, and JV participated in study design and coordination. BT, EV, and VS participated in data acquisition and maintained the database for analysis. EV and VS analyzed the data. AS, BT, CC, EV, HA, JV, SZ, and VS contributed to the critical revision of this work. All authors contributed to the article and approved the submitted version.

## Conflict of Interest

The authors declare that the research was conducted in the absence of any commercial or financial relationships that could be construed as a potential conflict of interest.

## References

[1] Madsen HO Garred P Thiel S Kurtzhals JA Lamm LU Ryder LP. Interplay between promoter and structural gene variants control basal serum level of mannan-binding protein. J Immunol. (1995) 155:3013–20. 7673719

[2] Turner MW. The role of mannose-binding lectin in health and disease. Mol Immunol. (2003) 40:423–9. 10.1016/S0161-5890(03)00155-X 14568388

[3] Kilpatrick DC. Mannan-binding lectin and its role in innate immunity. Transfus Med. (2002) 12:335–52. 10.1046/j.1365-3148.2002.00408.x 12473150

[4] Zhang D-F Huang X-Q Wang D Li Y-Y Yao Y-G. Genetic variants of complement genes ficolin-2, mannose-binding lectin and complement factor H are associated with leprosy in Han Chinese from Southwest China. Hum Genet. (2013) 132:629–40. 10.1007/s00439-013-1273-8 23423485

[5] Auriti C Prencipe G Moriondo M Bersani I Bertaina C Mondì V. Mannose-binding lectin: biologic characteristics and role in the susceptibility to infections and ischemia-reperfusion related injury in critically Ill neonates. J Immunol Res. (2017) 2017:7045630. 10.1155/2017/7045630 28246614 PMC5299167

[6] Teillet F Dublet B Andrieu J-P Gaboriaud C Arlaud GJ Thielens NM. The two major oligomeric forms of human mannan-binding lectin: chemical characterization, carbohydrate-binding properties, and interaction with MBL-associated serine proteases. J Immunol. (2005) 174:2870–7. 10.4049/jimmunol.174.5.2870 15728497

[7] Butler GS Sim D Tam E Devine D Overall CM. Mannose-binding lectin (MBL) mutants are susceptible to matrix metalloproteinase proteolysis potential role in human mbl deficiency. J Biol Chem. (2002) 277:17511–9. 10.1074/jbc.M201461200 11891230

[8] Ambrosio AR de Messias-Reason IJT. Leishmania (Viannia) braziliensis: interaction of mannose-binding lectin with surface glycoconjugates and complement activation. An antibody-independent defence mechanism. Parasite Immunol. (2005) 27:333–40. 10.1111/j.1365-3024.2005.00782.x 16149991

[9] de Miranda Santos IKF Costa CHN Krieger H Feitosa MF Zurakowski D Fardin B. Mannan-binding lectin enhances susceptibility to visceral leishmaniasis. Infect Immun. (2001) 69:5212–5. 10.1128/IAI.69.8.5212-5215.2001 11447210 PMC98624

[10] Areeshi MY Mandal RK Akhter N Dar SA Jawed A Wahid M. A meta-analysis of MBL2 polymorphisms and Tuberculosis risk. Sci Rep. (2016) 6:35728. 10.1038/srep35728 27876780 PMC5120291

[11] Garred P Harboe M Oettinger T Koch C Svejgaard A. Dual role of mannan-binding protein in infections: another case of heterosis? Eur J Immunogenet. (1994) 21:125–31. 10.1111/j.1744-313X.1994.tb00183.x 9098426

[12] Remus N Alcais A Abel L. Human genetics of common mycobacterial infections. Immunol Res. (2003) 28:109–29. 10.1385/IR:28:2:109 14610288

[13] Eichelmann K Gonzalez Gonzalez SE Salas-Alanis JC Ocampo-Candiani J. Leprosy. An update: definition, pathogenesis, classification, diagnosis, and treatment. Actas Dermosifiliogr. (2013) 104:554–63. 10.1016/j.adengl.2012.03.028 23870850

[14] Han XY Seo Y-H Sizer KC Schoberle T May GS Spencer JS. A new Mycobacterium species causing diffuse lepromatous leprosy. Am J Clin Pathol. (2008) 130:856–64. 10.1309/AJCPP72FJZZRRVMM 19019760

[15] Leprosy Available online at: http://www.who.int/en/news-room/fact-sheets/detail/leprosy (accessed August 15, 2018).

[16] Lastória JC Abreu M Hanseníase: diagnóstico e tratamento. Diagn Trat. (2012) 17:173–9.

[17] World Health Organization Global Health Observatory (GHO) data | Leprosy. WHO. World Health Organization (2019). Available online at: https://www.who.int/gho/neglected_diseases/leprosy/en/ (accessed October 13, 2019).

[18] de Messias-Reason IJ Boldt ABW Moraes Braga AC Von Rosen Seeling Stahlke E Dornelles L Pereira-Ferrari L. The association between mannan-binding lectin gene polymorphism and clinical leprosy: new insight into an old paradigm. J Infect Dis. (2007) 196:1379–85. 10.1086/521627 17922403

[19] Dornelles LN Pereira-Ferrari L Messias-Reason I Mannan-binding lectin plasma levels in leprosy: deficiency confers protection against the lepromatous but not the tuberculoid forms. Clin Exp Immunol. (2006) 145:463–8. 10.1111/j.1365-2249.2006.03161.x 16907914 PMC1809702

[20] Sapkota BR Macdonald M Berrington WR Misch EA Ranjit C Siddiqui MR. Association of TNF, MBL, and VDR polymorphisms with leprosy phenotypes. Hum Immunol. (2010) 71:992–8. 10.1016/j.humimm.2010.07.001 20650301 PMC2941523

[21] Probst CM Bompeixe EP Pereira NF de O Dalalio MM Visentainer JE Tsuneto LT. HLA polymorphism and evaluation of European, African, and Amerindian contribution to the white and mulatto populations from Paraná, Brazil. Hum Biol. (2000) 597–617. 11048789

[22] Steffensen R Thiel S Varming K Jersild C Jensenius JC. Detection of structural gene mutations and promoter polymorphisms in the mannan-binding lectin (MBL) gene by polymerase chain reaction with sequence-specific primers. J Immunol Methods. (2000) 241:33–42. 10.1016/S0022-1759(00)00198-8 10915847

[23] Barrett JC Fry B Maller J Daly MJ. Haploview: analysis and visualization of LD and haplotype maps. Bioinformatics. (2005) 21:263–5. 10.1093/bioinformatics/bth457 15297300

[24] Sole X Guino E Valls J Iniesta R Moreno V. SNPStats: a web tool for the analysis of association studies. Bioinformatics. (2006) 22:1928–9. 10.1093/bioinformatics/btl268 16720584

[25] Gauderman WJ. Sample size requirements for association studies of gene-gene interaction. Am J Epidemiol. (2002) 155:478–84. 10.1093/aje/155.5.478 11867360

[26] Pescarini JM Strina A Nery JS Skalinski LM Andrade KVF de Penna MLF. Socioeconomic risk markers of leprosy in high-burden countries: a systematic review and meta-analysis. PLoS Negl Trop Dis. (2018) 12:e0006622. 10.1371/journal.pntd.0006622 29985930 PMC6053250

[27] Vasconcelos LRS Fonseca JPL do Carmo RF de Mendonca TF Pereira VRA Lucena-Silva N. Mannose-binding lectin serum levels in patients with leprosy are influenced by age and MBL2 genotypes. Int J Infect Dis. (2011) 15:e551–7. 10.1016/j.ijid.2011.04.008 21640628

[28] Bonar A Chmiela M Rudnicka W Rozalska B. Mannose-binding lectin enhances the attachment and phagocytosis of mycobacteria in vitro. Arch Immunol Ther Exp Ed. (2005) 53:437. 16314827

[29] Callegaro-Filho D Shrestha N Burdick AE Haslett PA. A potential role for complement in immune evasion by Mycobacterium leprae. J Drugs Dermatol. (2010) 9:1373–82. 21061760

[30] Dallmann-Sauer M Correa-Macedo W Schurr E. Human genetics of mycobacterial disease. Mamm Genome. (2018) 29:523–38. 10.1007/s00335-018-9765-4 30116885 PMC6132723

