# Supplementary material for: Association of MBL2 Exon 1 Polymorphisms With Multibacillary Leprosy
Source: Front Immunol. 2020 Sep 3;11:1927. doi: 10.3389/fimmu.2020.01927 (PMC7494844; doi:10.3389/fimmu.2020.01927)
Supplement: Supplementary file 1 [file Data_Sheet_1.docx]

**Supplementary Tables**

**Table 1. Clinical and demographic profile of patients with leprosy and controls.**

| **Groups** | **Clinical form** | ***n*** | **Gender** | | **Age**  **Mean ± SD**  **(years)** | **Ethnic group** | |
| --- | --- | --- | --- | --- | --- | --- | --- |
|  |  |  | **Male (%)** | **Female (%)** |  | **Caucasoid**  **(%)** | **Others**  **(%)** |
| MB | Lepromatous or dimorph | 279 | 170 (60.93) | 109 (39.07) | 54.79 ± 12.74 | 174 (62.37) | 105 (37.63) |
| PB | Tuberculoid or indeterminate | 71 | 23  (32.39) | 48 (67.61) | 52.88 ± 11.76 | 50 (70.42) | 21 (29.58) |
| Controls |  | 350 | 180 (51.43) | 170 (48.57) | 56.24 ± 13.21 | 246 (70.29) | 104 (29.71) |

PB: paucibacillary; MB: multibacillary

**Table 2. MBL serum concentrations**

| **Sample** | **Leprosy’s**  **operational classification** | **Gender** | **Age** | **D variant** | **B variant** | **C variant** | **Grouped genotype** | **[MBL]**  **(ng/µl)** |
| --- | --- | --- | --- | --- | --- | --- | --- | --- |
| 1 | PB | F | 42 | C/C | G/G | G/G | A/A | 1316.55 |
| 2 | MB | M | 55 | C/C | G/A | G/G | A/O | 2892.72 |
| 3 | PB | M | 37 | C/C | G/G | G/G | A/A | 48.05 |
| 4 | PB | F | 50 | C/C | G/G | A/A | O/O | 954.29 |
| 5 | PB | F | 49 | C/C | G/G | G/G | A/A | 352.63 |
| 6 | MB | M | 80 | C/C | G/A | G/G | A/O | 2102.13 |
| 7 | PB | F | 58 | C/C | G/G | G/G | A/A | 1937.70 |
| 8 | MB | F | 51 | C/C | G/G | G/A | A/O | 658.02 |
| 9 | PB | M | 63 | C/T | G/G | G/G | A/O | 23.43 |
| 10 | MB | F | 45 | C/T | G/G | G/G | A/O | 3006.62 |
| 11 | MB | M | 79 | C/C | G/G | G/A | A/O | 1057.73 |
| 12 | PB | F | 44 | C/T | G/G | G/G | A/O | 3553.62 |
| 13 | PB | M | 75 | C/C | G/G | G/G | A/A | 2872.87 |
| 14 | MB | M | 53 | C/C | G/A | G/G | A/O | 590.68 |
| 15 | PB | M | 41 | C/C | G/G | G/G | A/A | 2040.11 |
| 16 | MB | M | 58 | C/C | G/A | G/G | A/O | 1640.12 |
| 17 | MB | M | 46 | C/C | G/A | G/G | A/O | 281.04 |
| 18 | PB | F | 61 | C/C | G/A | G/G | A/O | 3349.49 |
| 19 | PB | F | 66 | C/C | G/G | G/G | A/A | 1989.97 |
| 20 | PB | M | 53 | C/C | G/G | G/A | A/O | 378.16 |
| 21 | MB | M | 49 | C/T | G/A | G/G | O/O | 207.45 |
| 22 | PB | M | 71 | C/T | G/G | G/G | A/O | 3220.07 |
| 23 | MB | M | 33 | C/C | G/A | G/G | A/O | 2500.91 |
| 24 | MB | F | 59 | C/C | G/G | G/G | A/A | 433.74 |
| 25 | PB | F | 41 | C/C | G/G | G/G | A/A | 214.19 |
| 26 | MB | M | 46 | C/C | G/A | G/G | A/O | 736.33 |
| 27 | MB | F | 55 | C/C | G/G | G/G | A/A | 61.00 |
| 28 | MB | M | 55 | C/C | G/G | G/A | A/O | 519.05 |
| 29 | MB | M | 32 | C/C | A/A | G/G | O/O | 2857.57 |
| 30 | PB | F | 50 | C/C | G/G | G/G | A/A | 1409.46 |
| 31 | PB | F | 74 | C/C | G/G | G/G | A/A | 38.32 |
| 32 | MB | F | 32 | C/C | G/A | G/G | A/O | 278.39 |
| 33 | MB | M | 57 | C/T | G/G | G/A | O/O | 1724.23 |
| 34 | PB | F | 73 | C/C | G/G | G/G | A/A | 383.60 |
| 35 | MB | M | 43 | C/C | G/A | G/G | A/O | 4620.02 |
| 36 | MB | M | 53 | C/C | G/G | G/A | A/O | 2660.37 |
| 37 | MB | F | 37 | C/T | G/G | G/G | A/O | 529.28 |
| 38 | MB | F | 51 | C/C | G/G | G/G | A/A | 803.11 |
| 39 | PB | M | 48 | C/C | G/G | G/G | A/A | 1802.44 |
| 40 | PB | F | 51 | C/C | G/A | G/G | A/O | 2125.76 |
| 41 | PB | F | 39 | C/T | G/G | G/G | A/O | 15.60 |
| 42 | MB | F | 55 | C/C | G/G | G/A | A/O | 1063.51 |
| 43 | PB | M | 53 | C/T | G/G | G/G | A/O | 3775.05 |
| 44 | MB | M | 64 | C/C | A/A | G/G | O/O | 47.74 |
| 45 | MB | F | 50 | C/C | G/G | G/G | A/A | 2325.15 |
| 46 | PB | F | 67 | T/T | G/G | G/G | O/O | 16.24 |
| 47 | MB | F | 44 | C/C | G/A | G/G | A/O | 290.61 |
| 48 | PB | F | 51 | C/C | G/G | G/G | A/A | 2610.30 |
| 49 | MB | F | 58 | C/C | G/G | G/G | A/A | 3435.79 |
| 50 | PB | F | 41 | C/C | G/A | G/G | A/O | 302.47 |
| 51 | PB | M | 52 | C/C | G/G | G/G | A/A | 2289.68 |
| 52 | MB | F | 53 | C/C | G/G | G/A | A/O | 1187.21 |
| 53 | MB | F | 67 | C/C | G/A | G/G | A/O | 60.51 |
| 54 | PB | F | 63 | C/C | G/G | G/G | A/A | 4484.02 |
| 55 | MB | M | 57 | C/C | G/G | G/G | A/A | 5613.07 |
| 56 | PB | F | 72 | C/C | G/G | G/G | A/A | 1782.27 |
| 57 | PB | M | 68 | C/C | G/G | G/G | A/A | 3435.79 |
| 58 | MB | M | 45 | C/C | G/G | A/A | O/O | 15.89 |
| 59 | MB | M | 79 | C/C | G/G | G/A | A/O | 23.97 |
| 60 | PB | M | 46 | C/C | G/G | G/A | A/O | 2095.90 |
| 61 | MB | F | 61 | C/C | G/A | G/G | A/O | 477.98 |
| 62 | PB | F | 60 | C/C | G/G | G/G | A/A | 1289.21 |
| 63 | MB | F | 77 | C/C | A/A | G/G | O/O | 18.02 |
| 64 | MB | M | 65 | C/C | G/G | G/A | A/O | 570.69 |
| 65 | PB | F | 58 | C/C | G/G | G/G | A/A | 4774.66 |
| 66 | PB | F | 37 | C/C | G/A | G/G | A/O | 231.50 |
| 67 | MB | F | 66 | C/T | G/G | G/G | A/O | 41.57 |
| 68 | MB | M | 61 | C/C | G/G | G/G | A/A | 2107.74 |
| 69 | MB | M | 36 | C/C | G/G | G/G | A/A | 3266.02 |
| 70 | PB | F | 50 | C/C | G/A | G/G | A/O | 217.78 |
| 71 | MB | M | 50 | C/C | G/G | G/G | A/A | 3882.81 |
| 72 | MB | M | 65 | C/C | G/A | G/G | A/O | 70.14 |
| 73 | MB | M | 55 | C/C | G/G | G/G | A/A | 2075.74 |
| 74 | MB | M | 68 | C/C | G/G | G/G | A/A | 3766.12 |
| 75 | PB | M | 33 | C/C | G/A | G/G | A/O | 306.39 |
| 76 | MB | M | 71 | C/C | A/A | G/G | O/O | 15.96 |
| 77 | MB | M | 67 | C/T | G/G | G/G | A/O | 3984.72 |
| 78 | MB | M | 60 | C/C | G/A | G/G | A/O | 122.88 |
| 79 | PB | F | 52 | C/T | G/G | G/G | A/O | 24.04 |
| 80 | MB | F | 42 | C/C | G/G | A/A | O/O | 17.53 |

PB: paucibacillary; MB: multibacillary; F: female; M: male
